# Advanced lesions of synchronous bilateral mammary Paget’s disease: a case report 

**DOI:** 10.1186/s13256-020-02442-5

**Published:** 2020-08-04

**Authors:** Lina Choridah, Wida Kartika Sari, Ery Kus Dwianingsih, Irianiwati Widodo, Suwardjo, Sumadi Lukman Anwar

**Affiliations:** 1 Department of Radiology, Dr Sardjito Hospital/Faculty of Medicine, Public Health, and Nursing, Universitas Gadjah Mada, Yogyakarta, 55281 Indonesia; 2 Department of Anatomical Pathology, Dr Sardjito Hospital/Faculty of Medicine, Public Health, and Nursing, Universitas Gadjah Mada, Yogyakarta, 55281 Indonesia; 3 Division of Surgical Oncology – Department of Surgery – Dr Sardjito Hospital/Faculty of Medicine, Public Health, and Nursing, Universitas Gadjah Mada, Jl Kesehatan No. 1, Yogyakarta, 55281 Indonesia

**Keywords:** Paget’s disease, Breast cancer, Areolae eczema, Bilateral synchronous, Delayed diagnosis

## Abstract

**Background:**

Mammary Paget’s disease is an eczematous eruption on the nipple and areola with underlying breast malignancy. It is often misinterpreted as chronic dermatitis or psoriasis causing a delayed diagnosis. Synchronous bilateral mammary Paget’s disease is exceptionally rare and an advanced case with underlying invasive carcinoma might require long-term treatment and follow-up that could affect a patient’s physical, psychological, and social aspects of well-being.

**Case presentation:**

A 54-year-old Javanese woman presented in our clinic with a 2-year history of itching and chronic eczema in both areolae. Bilateral nipple retraction and retro-areolar palpable lumps were observed during the first presentation. Breast ultrasound revealed hypoechoic lesions in her left and right breasts. Mammograms showed an irregular hyperdense lesion and multiple microcalcifications. Histopathology from biopsy and bilateral mastectomy demonstrated infiltration of large Paget’s cells in the epidermis of the areola with underlying lesions of invasive ductal carcinoma, diagnosed solid type with high nuclear grade and negative expression of estrogen receptor and progesterone receptor, with positive expression of human epidermal growth receptor-2(HER2) and Ki-67 (45%).

**Conclusions:**

In a patient with suspicious chronic inflammation of the nipple and areolae, prompt biopsy should be performed to avoid a delayed diagnosis of any malignant breast lesion.

## Background

Mammary Paget’s disease presents as an eczema-like skin rash, with itching, ulceration, or scaly changes affecting the nipple and areola; it is frequently found with an underlying malignant tumor [1]. The disease is often incorrectly assumed to be atopic or contact dermatitis, psoriasis, or other benign dermatological lesions [1]. Around 90% of patients are diagnosed without a palpable mass during physical examination [1, 2]. Diagnosis is established through full-thickness skin biopsy showing the invasion of epidermis by typical giant glandular epithelial cells with hyperchromatic nuclei known as Paget’s cells [1, 2]. The diagnosis is frequently developed through clinical findings; however, radiological imaging is required to detect the underlying carcinomas and to determine clinical staging for planning the further management [1, 2].

Paget’s disease represents 1–3% of all breast cancers and the synchronous bilateral case is very uncommon [1, 2]. In patients with an associated palpable mass, the concurrent tumor is likely to be an invasive carcinoma located in the center of the breast with axillary lymph node infiltration [2]. Therefore, patients with mammary Paget’s disease with evidence of palpable mass most probably require both radical surgery and adjuvant treatment [3, 4]. Because the initial appearance resembles benign skin lesions, diagnosis of mammary Paget’s disease is often delayed [3–5]. In low-income and middle-income countries, including Indonesia, the delay in diagnosis is even longer due to the relatively low level of cancer awareness and limited health facilities [6, 7]. In a case of bilateral Paget’s disease with associated invasive carcinomas and lymph node infiltration, bilateral mastectomy and subsequent adjuvant local and systemic treatment remain the preferred treatment choice [3, 4]. In addition, positive family history and genetic predisposition are independent risk factors of synchronous bilateral breast cancer [8]. Therefore, psychosocial support and multidisciplinary rehabilitation are highly recommended to promote emotional and behavioral adaptation after the acute treatment. We reported a case of synchronous bilateral mammary Paget’s disease who underwent bilateral MRMs.

## Case presentation

**Fig. 1:**
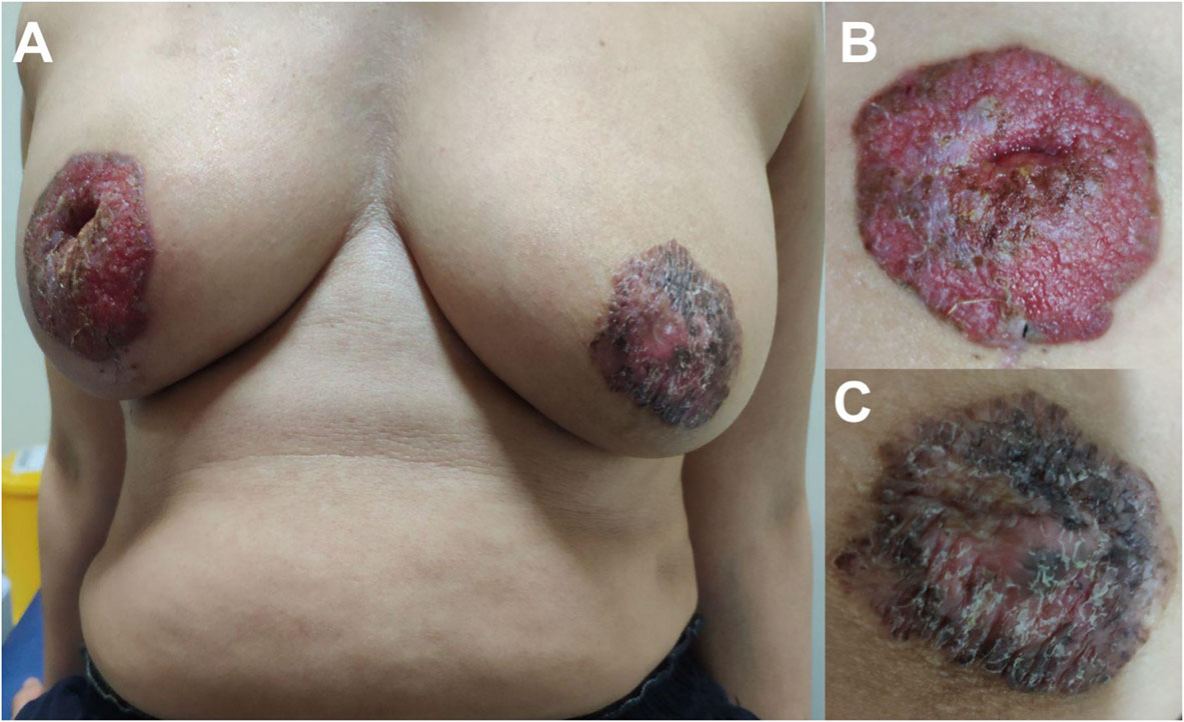
Clinical presentation at diagnosis. **a** Clinical image showing synchronous bilateral mammary Paget’s disease. **b** Right nipple-areolae complex showing ulceration in the central area and an eczematous scaly lesion. **c** Left nipple-areolae complex showing an eczematous, pigmented, scaly lesion

**Fig. 2:**
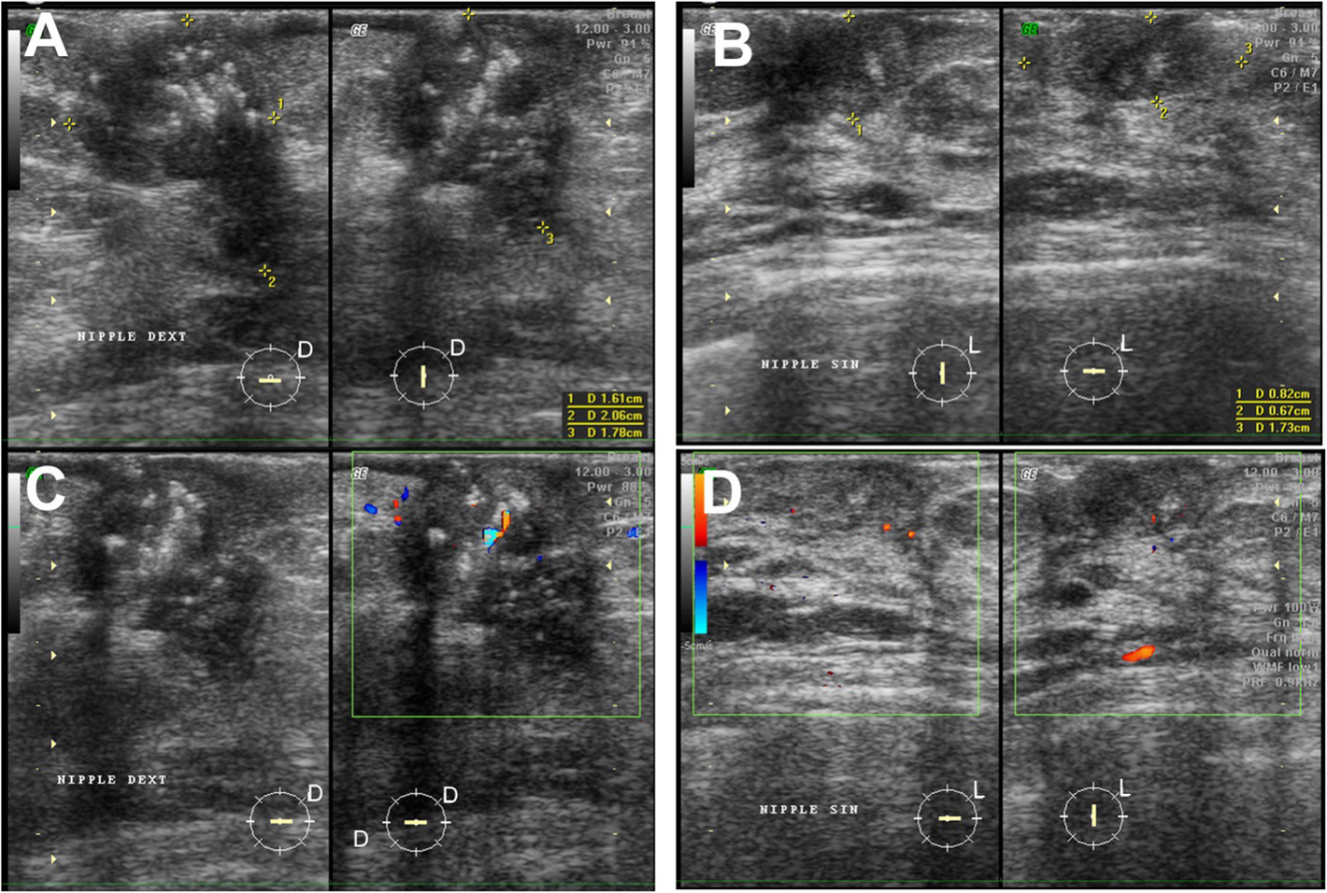
Ultrasound images showing hypoechoic lesion with calcification measuring around 1.61 × 2.06 × 1.78 cm at right retro-papilla (**a**) and an amorphic, irregular lesion measuring 0.82 × 0.67 × 1.73 cm located at the left retro-papilla (**b**) with relative hypervascularization at the right and left nipple-areolae complexes (**c**, **d**, respectively)

**Fig. 3:**
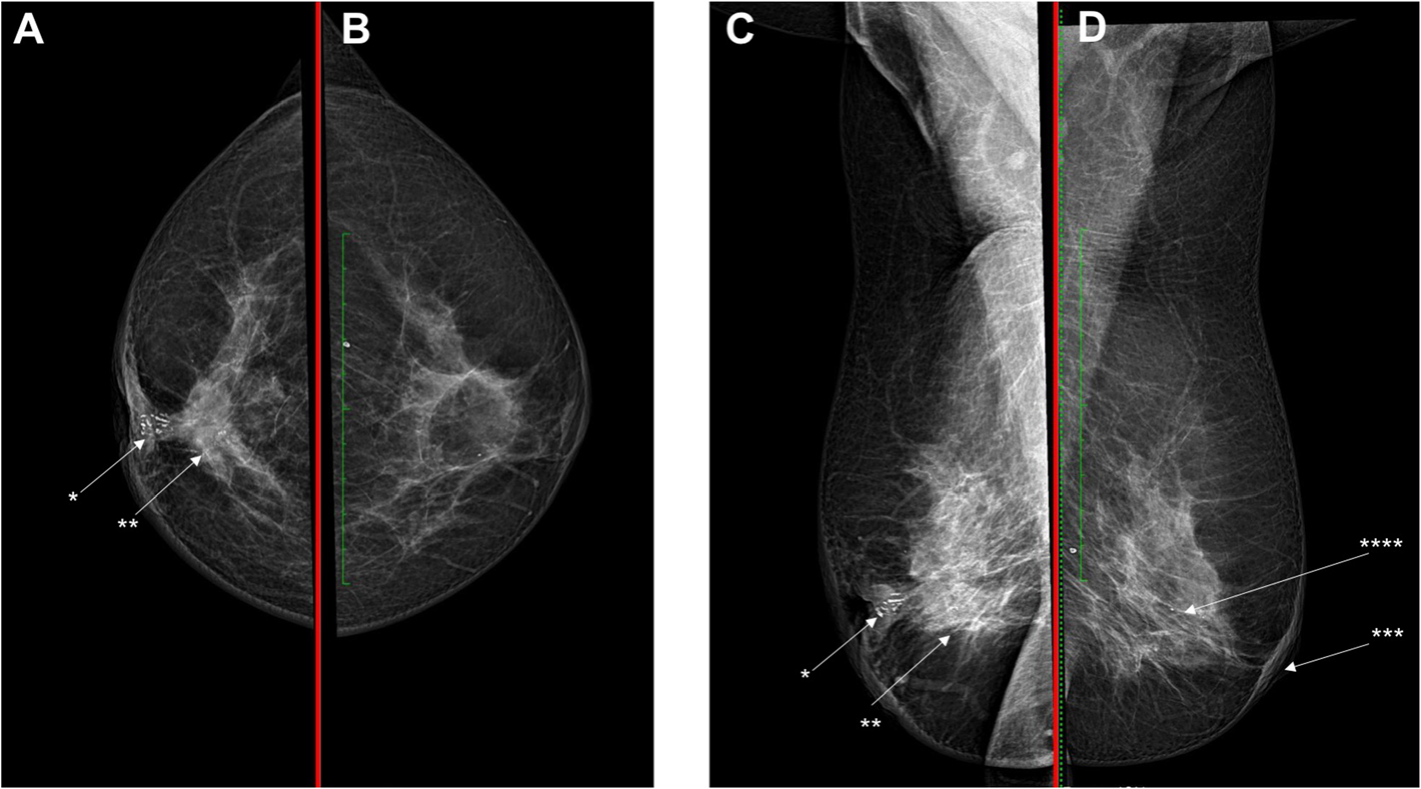
Images of mammograms (**a**, **b** craniocaudal views; **c**, **d** mediolateral oblique views). Mammograms of the right breast (**a** and **c**) show an amorphic hyperdense lesion measuring around 1.26 × 2.12 cm which was centrally located approximately 1.75 cm from the right nipple (**) and multiple microcalcifications (*). Mammograms of the left breast (**b** and **d**) show skin thickening (***) and retro-papillae multiple microcalcifications (****)

**Fig. 4:**
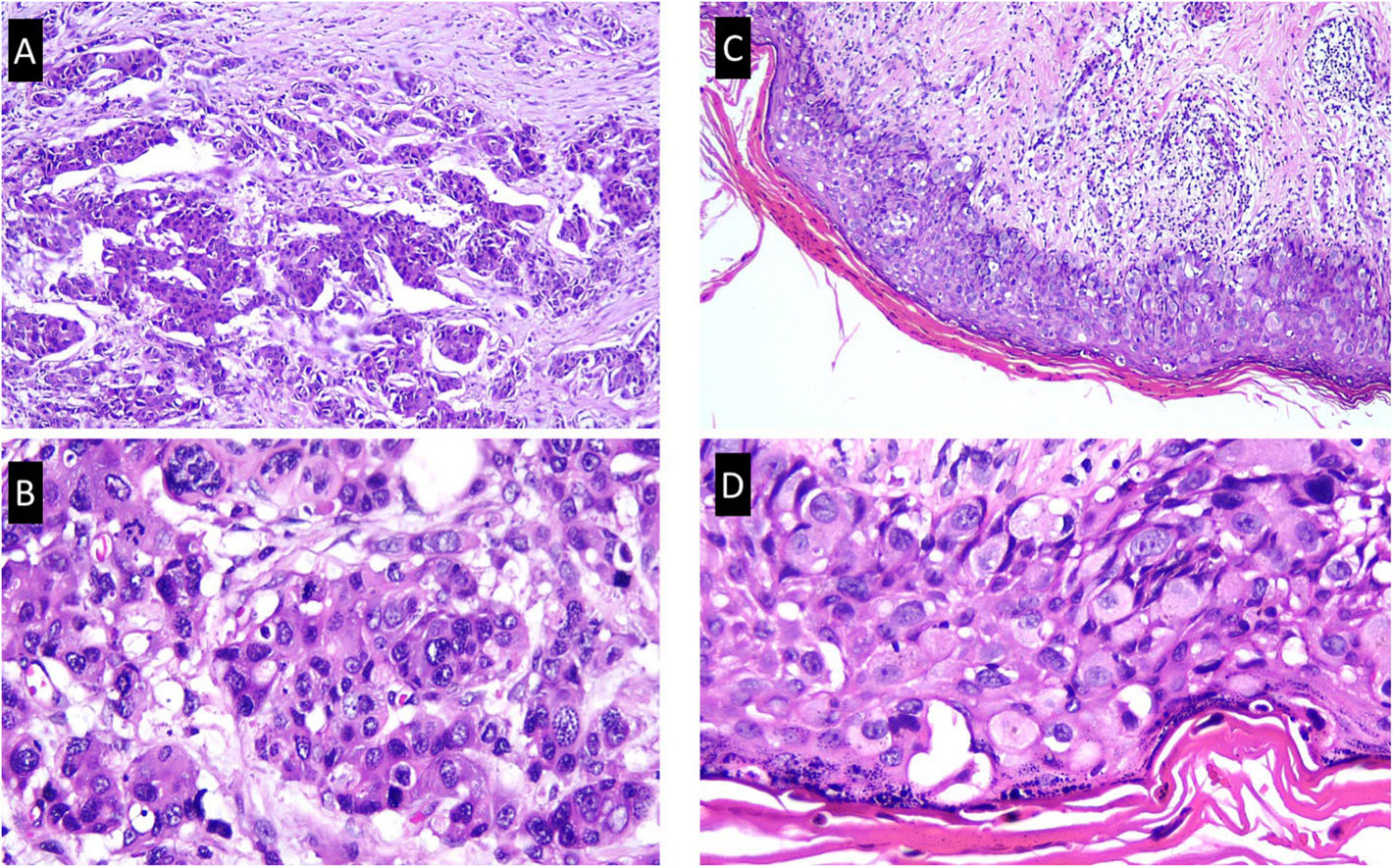
Nests of tumor cells, infiltrating surrounding fibrous tissue (hematoxylin and eosin staining). **a** Tumor nests of the main breast mass (× 100 magnification]. **b** Tumor cells are pleomorphic, large size, with vesicular and hyperchromatic nuclei (× 200 magnification). **c** Skin epidermis is infiltrated by numerous tumor cells which resemble the main lesion (× 100 magnification). **d** Characteristics of the tumor cells are similar to **b** (× 200 magnification)

**Fig. 5:**
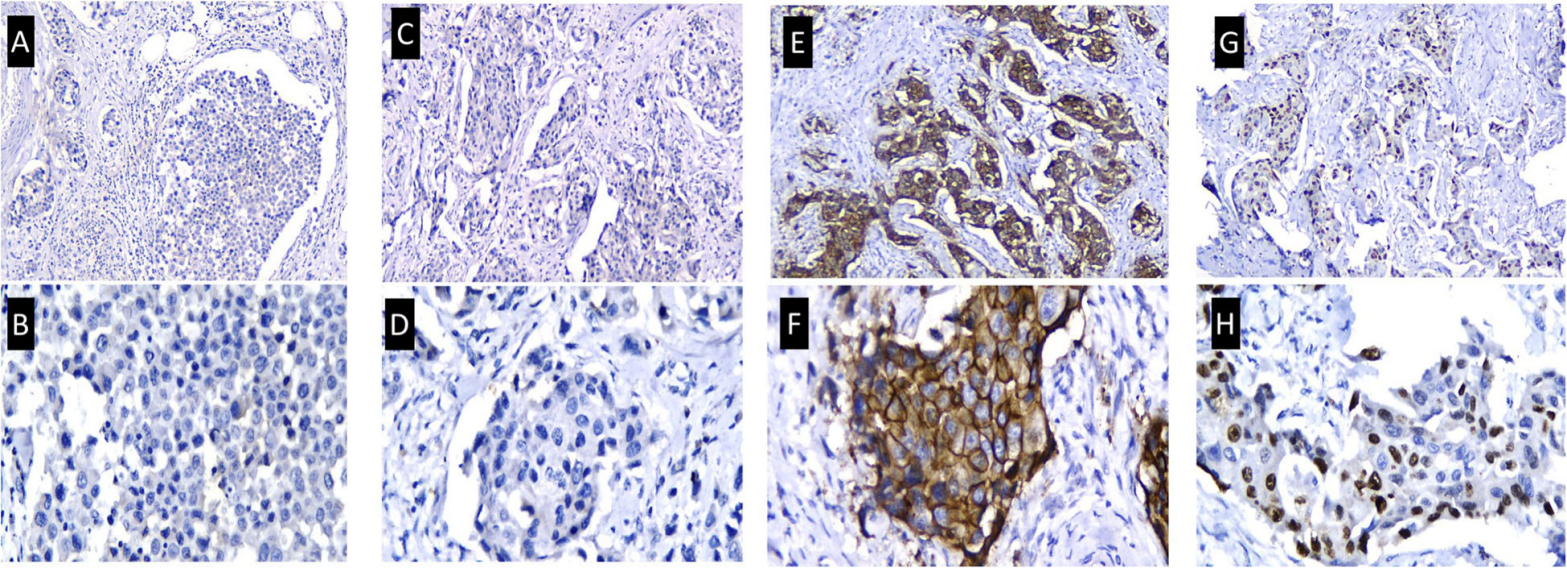
Immunostaining images of estrogen receptor, progesterone receptor, human epidermal growth receptor-2, and Ki-67 expression. **a**, **b** Tumor cells are negatively stained with estrogen receptor in the nuclei (× 100 magnification and × 200 magnification). **c**, **d** Tumor cells are negatively stained with progesterone receptor in the nuclei (× 100 magnification and × 200 magnification). **e**, **f** Tumor cells showed strong membrane expression of human epidermal growth receptor-2 (× 100 magnification and × 200 magnification). **g**, **h** Strong nuclear positivity of Ki-67 in > 20% of tumor cells (× 100 magnification and × 200 magnification)

A 54-year-old Javanese woman presented to our hospital with a 2-year history of non-healing scaly rash and itching in both nipples and areolae without any family history of breast or ovarian cancer. She was previously examined by general practitioners as well as a dermatologist and received anti-allergy topical treatment before undergoing a biopsy. On physical examination, eczema, excoriation, and ulceration of both nipples and areola were observed with right nipple retraction and no obvious nipple discharge (Fig. 1). Irregular firmly formed retro-papilla lumps of size 2 × 2 cm and 1 × 2 cm were palpated in her right and left breasts, respectively. No palpable axillary lymph node was observed in the bilateral axillae. Ultrasonography of both breasts showed a spiculated hypoechoic lesion with calcification measuring around 1.61 × 2.06 × 1.78 cm in the right retro-papilla and an amorphic, irregular lesion measuring 0.82 × 0.67 × 1.73 cm located in the left retro-papilla (Fig. 2). Both lesions were categorized as Breast Imaging Reporting and Data System (BIRADS) IV (Fig. 3). Mammograms showed an amorphic hyperdense lesion measuring around 1.26 × 2.12 cm that was centrally located approximately 1.75 cm in the right nipple and multiple retro-papillae microcalcifications in her left breast (Fig. 3). A histopathological examination showed typical Paget’s disease of the bilateral breasts with underlying invasive ductal carcinoma, diagnosed as high grade with lymphovascular invasion (Fig. 4). Pathological staging was pT2 pN1 cM0 for the right breast and pT1 pN0 cM0 for the left breast. Immunostaining showed negative expression of estrogen receptor (ER) and progesterone receptor (PR) and positive expression of human epidermal growth receptor-2 (HER2) and Ki-67 (45%) (Fig. 5). Our patient underwent bilateral modified radical mastectomies (MRMs) and completed the self-reported questionnaires of Indonesian adapted European Organisation for Research and Treatment of Cancer (EORTC) Quality of Life questionnaire (QLQ)-C30 [9] and QLQ-BR23.

## Discussion

Mammary Paget’s disease is a rare clinical pathology of the nipple and areolae and often presents in conjunction with an underlying breast carcinoma [1, 10]. Early manifestation might present as scaling or redness that is often misinterpreted as eczema or chronic inflammatory reaction causing a delayed diagnosis [1, 11]. Round plaque with eczema-like rash and ulceration limited to the areolae with or without bloody/serous discharge represent an advanced lesion of Paget’s disease [1, 11]. It is often accompanied by inversion or retraction of the nipple. A unilateral lesion is mostly found whereas the synchronous bilateral case is very uncommon. Several benign skin lesions that often cause misinterpretation of mammary Paget’s disease as well as common clinical manifestations that can be used to differentiate it from benign inflammatory skin lesions are summarized in Table 1. We report the case of a patient with advanced lesions of bilateral mammary Paget’s disease and concomitant palpable masses (Figs. 1, 2 and 3). Delayed diagnosis is associated with multiple factors including patient delay (lack of knowledge and awareness, use of traditional therapy, fear of treatment, financial constraints, competing life priority, and embarrassment of breast cancer diagnosis) and system delay (complicated referral system, appointment delay, misdiagnosis, misinterpretation of mammography or diagnostic tests, and limited health facilities) [12]. Both factors might have influenced the delayed diagnosis in our case, therefore, further study is required to identify determinants causing delayed diagnosis of breast cancer in Indonesia.

**Table 1:** Benign skin and mammary lesions that often cause misinterpretation with mammary Paget’s disease

Differential diagnosis of mammary Paget’s disease	Clinical manifestations of mammary Paget’s disease and benign inflammatory skin lesions in the nipple	
	Paget’s disease	Benign inflammatory skin lesions in the nipple
Eczema	Not itchy or slight itchy and is non-responsive to treatment	Itchy
Psoriasis	No vesicles or pustules	Vesicles and pustules
Irritant contact dermatitis	Nipple retraction or deformation	No change in the nipple, limited in areolae lesions
Mammary duct ectasia	Usually unilateral	Bilateral
Drug eruption	Sometimes with palpable lump	No lump
Toker cells	Older age	Younger age
Nipple duct adenoma	Mammographic lesions/microcalcifications	Normal mammogram

The second column lists some clinical manifestations that can be used to differentiate benign nipple areola inflammation from mammary Paget’s disease

Any suspected Paget’s disease should be further assessed with radiological imaging because of its high association with *in situ* or invasive breast carcinoma [13]. Imaging is very important to determine the suitable option for surgery as well as the adjuvant treatment [13]. Breast ultrasonography is useful particularly if it is used in combination with mammography [14]. Breast ultrasonography is able to detect any hypoechoic area, discrete mass, heterogeneous parenchyma, skin thickening, and dilated ducts which are mostly nonspecific findings with signs of infection [14]. In addition, bilateral mammograms are very important to detect an underlying lesion including a mass or cluster of microcalcifications, to exclude multifocal carcinomas, as well as to evaluate the contralateral breast. Other presentations of mammary Paget’s disease are thickening of skin, nipple, and areolae as well as nipple retraction, subareolar microcalcification, discrete mass, and architectural distortion [5, 13–15]. Although a mammogram is not always able to detect the underlying ductal carcinoma *in situ* (DCIS), the sensitivity is particularly higher in patients with palpable mass [13]. We revealed underlying tumors in the bilateral breasts using sonography and mammography in a patient with synchronous bilateral Paget’s disease (Figs. 2 and 3). Breast magnetic resonance imaging (MRI) is considered superior to detect the underlying malignancy in Paget’s disease because of its ability to better demonstrate abnormal enhancement or thickening of the nipple-areolar complex, as well as an enhancing *in situ* or invasive tumor including in a clinically unpalpable mass [13]. However, MRI is not always widely available in low-income and middle-income countries and referral for MRI examination is usually made only in occult disease after careful assessment of clinical and mammogram evaluations.

In Paget’s disease, the main characteristic of histopathological examination is the presence of intraepidermal Paget’s cells, which are glandular epithelial cells with a large clear cytoplasm and enlarged hyperchromatic nuclei that are often found in a basal layer without any intercellular bridges to the adjacent cells [1, 2]. Expression levels of hormonal receptors, HER2, and Ki-67 vary among cases [1, 2]. Expression of ER and PR was negative and expression of HER2 was positive in our reported case. HER2 protein expression is associated with the capability to promote cell motility and intraepithelial spread [16]. In addition, the patient might be eligible for future therapy targeted therapy to HER2.

Surgery remains the mainstay of mammary Paget’s disease treatment. Mastectomy or subcutaneous mastectomy with or without axillary dissection has been practiced as a standard procedure. However, the current trend has shown the increased implementation of breast-conserving therapy for Paget’s disease with comparable clinical outcomes particularly in the cases with DCIS or without underlying tumor [3]. With the availability of sentinel node biopsy and radiotherapy, conservation surgery for mammary Paget’s disease has gained more interest [3]. In our case, we performed bilateral MRM because of the underlying invasive carcinomas in the bilateral breasts, unavailability of sentinel node biopsy, and potential long queue for radiotherapy. However, double mastectomies might result in a permanent change of appearance and body image of a woman. As the breasts represent femininity, beauty, motherhood, and women’s identity in Indonesian and many other cultures, the perceived loss might cause negative impacts on both physical and psychosocial aspects of well-being. Quality of life measurement using a self-reported questionnaire showed that our patient had lower scores in the global health status, and cognitive and social functioning domains compared to the reference scores of patients with breast cancer [17]. In addition, our patient had lower scores in future perspective and sexual functioning but higher scores in the symptom scales compared to the references [18]. Although several factors contribute to a patient’s quality of life score, including social-economic status, education, and the method of measurement, a self-reported questionnaire enables quick detection of an individual’s response to treatment and current state of well-being. Meeting the psychosocial needs of a patient over the course of treatment and surveillance is recommended to improve the patient’s acceptance, adherence to therapy, as well as their overall quality of life and well-being.

## Conclusion

Although the presentation of mammary Paget’s disease is rare, any suspected signs or symptoms of eczematoid, pigmented, crusted, or scaly lesions or chronic inflammation in the nipple or areolae should be confirmed with biopsy to avoid delayed diagnosis. In particular, in Indonesia, further public health actions are therefore required to improve breast cancer awareness as well as to strengthen the current health system for better breast cancer management.

## Data Availability

The clinical and imaging data supporting the analysis and findings of this study will be available from the corresponding author upon reasonable request.

## Abbreviations

BIRADS: Breast Imaging Reporting and Data System
DCIS: Ductal carcinoma *in situ*
ER: Estrogen receptor
EORTC: European Organisation for Research and Treatment of Cancer
HER2: Human epidermal growth receptor-2
MRI: Magnetic resonance imaging
MRM: Modified radical mastectomy
PR: Progesterone receptor
QLQ: Quality of Life questionnaire
